# Adolescent substance use and peer use: a multilevel analysis of cross-sectional population data

**DOI:** 10.1186/1747-597X-8-27

**Published:** 2013-07-31

**Authors:** Alfgeir Logi Kristjansson, Inga Dora Sigfusdottir, John P Allegrante

**Affiliations:** 1 West Virginia University, Morgantown, WV, USA; 2 Icelandic Centre for Social Research and Analysis, Reykjavik University, Reykjavik, Iceland; 3 Teachers College, Columbia University, New York, USA; 4 Mailman School of Public Health, Columbia University, New York, USA; 5 Department of Social and Behavioral Sciences, West Virginia University, School of Public Health, Robert C. Byrd Health Sciences Center, P.O. Box 9000, Morgantown 26506, WV, USA

**Keywords:** Adolescents, Context, Iceland, Multilevel, Substance use

## Abstract

**Background:**

Limited evidence exists concerning the importance of social contexts in adolescent substance use prevention. In addition to the important role schools play in educating young people, they are important ecological platforms for adolescent health, development and behaviors. In this light, school community contexts represent an important, but largely neglected, area of research in adolescent substance use and prevention, particularly with regard to peer influences. This study sought to add to a growing body of literature into peer contexts by testing a model of peer substance use simultaneously on individual and school community levels while taking account of several well established individual level factors.

**Method:**

We analyzed population-based data from the 2009 *Youth in Iceland* school survey, with 7,084 participants (response rate of 83.5%) nested within 140 schools across Iceland. Multilevel logistic regression models were used to analyze the data.

**Results:**

School-level peer smoking and drunkenness were positively related to adolescent daily smoking and lifetime drunkenness after taking account of individual level peer smoking and drunkenness. These relationships held true for all respondents, irrespective of socio-economic status and other background variables, time spent with parents, academic performance, self-assessed peer respect for smoking and alcohol use, or if they have substance-using friends or not. On the other hand, the same relationships were not found with regard to individual and peer cannabis use.

**Conclusions:**

The school-level findings in this study represent context effects that are over and above individual-level associations. This holds although we accounted for a large number of individual level variables that studies generally have not included. For the purpose of prevention, school communities should be targeted as a whole in substance use prevention programs in addition to reaching to individuals of particular concern.

## Introduction

“Although the effects of peer groups on adolescent substance use have been widely documented, much remains to be learned, especially regarding the mechanisms of peer influence” [1]. In a newly published review, this assertion signifies the current debate into the processes underlying peer influences in adolescent substance use. Peer substance use has long been shown to be of much influence in the development of individual use [2,3]. Much of the current literature divides peer influences in merely two categories: *socialization* and *selection*. Simplified, the former states that adolescents are socialized by the behavior, ideas, and norms of their peers, whereas the latter argues that adolescents select their friends consistent with their interests, seeking out those whom they perceive to behave similar to them and have similar norms and ideas about life [4-6]. Over time researchers have become increasingly convinced that both socialization and selection are important and that influences from one to the other and across are knitted into a complicated web of interrelations where no one factor fully predicts another, and that these components are often bidirectional [7-9]. While many studies have employed longitudinal models to test the development of socialization and selection in adolescent substance use e.g., [10-13], another aspect of peer influences has received much less attention, namely the one due to context.

In order to disentangle the relative associations of individual and contextual relations, a nested data structure is needed where they can be separated in a combined model without jeopardizing important statistical assumptions of significance tests [14]. Part of the explanation for the scarcity of such studies is the number of responses needed for the analysis to be methodologically possible. For this reason most longitudinal studies with repeated measures and nested data structures do not incorporate measures aggregated to a higher level but focus more on variation in responses within individuals, which is a special class of nested models called growth curve models [15]. Although called for in review studies several years ago [16,17], studying higher level context and individual-level effects jointly is still, to date, relatively uncommon in research on adolescent substance use and peer use.

Adolescents spend a great deal of time inside and around their schools where they are directly and indirectly affected by school and community norms and culture in addition to the educational aspect of schools. Self-evidently, schools are important platforms in community substance use prevention and health promotion [18-20]. Despite the importance of school communities, not many studies have examined the impact of school community-level rates of peer use on individual use when also including measures for peer use on the individual level and other important covariates of adolescent substance use. Exceptions are studies by Leatherdale et al. [6,21] in which the authors found that higher school-level prevalence of smoking among the senior population contributed to an increase in odds for ever smoking in the junior population over schools with lower prevalence rates of senior students smoking. Authors also found a cross-level interaction between school-level senior student smoking rates and individual-level number of smoking friends. Kuntsche and Jordan [22] also found a strong association between substance using peers and adolescent drunkenness and cannabis use. In a separate study, Kuntche [23] found a contextual relationship between cannabis using peers and student’s cannabis use in classes where students saw others entering schools while cannabis-intoxicated or using cannabis substances on school premises. Also, Ennett et al., [24] found that adolescents bound to peer networks with higher smoking prevalence rates are more likely to smoke cigarettes and use marijuana than those with less ties to such networks. Other studies have examined country characteristics and adolescent substance use and found those to be of importance over and above individual level relationships [25]. These kinds of analyses are exception to the rule but provide important and novel insight into the environmental and cultural relations adolescents are subject to in their communities that are beyond and additive to their individual experiences. In line with such findings, many studies on adolescents conclude that substance use prevention should focus both at individual and group levels e.g., [26,27].

Based on the literature cited above and to further develop our understanding of the peer context, we used two-level nested data to test the hypothesis that individual- and school-level measures of peer substance use are positively related to daily smoking, lifetime drunkenness, and lifetime cannabis use among 14 to 16-year-old adolescents, and simultaneously on these two levels, among individuals and within school-communities. In our models we also take account of background factors, demographic and SES variables, parental preventive factors, academic performance, as well as perceived peer respect for substance use. We therefore suggest that aggregated school community level peer use positively associates with the odds of individual level daily smoking, lifetime drunkenness and lifetime cannabis use, suggesting contextual effects that are over and beyond individual-level relationships when controlling for other known risk and protective factors.

## Methods

### Sample

This study utilized population-wide, cross-sectional data from the latest in the series of surveys called *Youth in Iceland*, which monitor trends in a wide range of demographic, behavioral, and health-related variables [18]. Conducted by the Icelandic Centre for Social Research and Analysis (ICSRA), under the auspices of the Icelandic Ministry of Education, Science, and Culture, the survey on which we report was conducted during February of 2009 and included students aged 14 to 16 years who were enrolled in the 9^th^ (14 and 15 years of age) and 10^th^ (15 and 16 years of age) grades in nearly all Icelandic secondary schools. All aspects of data collection, including participant involvement based on passive parental consent, were conducted in accordance with Icelandic guidelines for the protection of research subjects.

Under ICSRA oversight, teachers at each school supervised questionnaire completion onsite. All students who attended school on the day that the survey was scheduled completed the questionnaires within their regular classrooms. Students were instructed not to write their name, social security number, or any other identifying information anywhere on the questionnaire booklet. They were instructed to complete the entire booklet, but to ask for help if they had any problems or any questions for clarification. Once students completed the questionnaires, they were asked to place their completed booklet in an envelope provided for that purpose, and seal the envelope before returning it to the supervising teacher. The total number of responses was 7,514 (50.8% girls) and yielded a response rate of 83.5% of the total national population in these age groups. A background check on the within-school non-response was carried out and revealed no particular pattern or bias in responses between schools. Due to item non-response on nominal variables such as gender, school, and moving house during last 12 months, where replacing values with their grand mean is not appropriate, data from 7,084 individuals that are nested within 140 schools were used in the current analysis. A background check concerning the deleted items was carried out and they appeared randomly distributed across the response groups. The missing values in continuous items were in all cases less than 2.4% of the total number of responses for each variable. Given this low frequency in missing information these data were replaced with the respective grand mean on each occasion. The number of valid responses within the 140 schools ranges from 1 to 275, with 23 schools having fewer than 10 participants (16.4%).

### Variables and measures

Approximately 90% of the estimated 320,000 inhabitants of Iceland are of Norse-Celtic decent, with 80% of the population belonging to the Lutheran State Church and no other religious institution having more than 3% of the population registered in its services [28]. Because of this homogeneity, exogenous variables such as race and religion, which are often used in research in other countries, were not included in the present analysis. Furthermore, enrolment in schools in Iceland is exclusively based on the inhabitants’ geographical area. Although exemptions do occur, the Icelandic school communities are defined by regional areas and town districts. As a result our school level data should contain the specific community characteristics for a given school community. Generally variables follow a normal distribution with a skew and kurtosis within the range of +/− 1.0 except those explained otherwise. Table 1 shows the descriptive statistics for all study variables.

**Table 1:** Descriptive statistics for all study variables (N = 7084)

***Categorical variables***	**Min**	**Max**	**n**	**%**
Daily smoking	0.00	1.00	470	6.6
Lifetime drunkenness	0.00	1.00	1986	28.0
Lifetime cannabis use	0.00	1.00	544	7.7
Peer cannabis use	0.00	1.00	1785	25.2
Girls	0.00	1.00	3648	51.5
Family structure (other)	0.00	1.00	2107	29.7
Attends school in home neighborhood	0.00	1.00	6139	86.7
Changed schools in last 12 months	0.00	1.00	490	6.9
Residential moving in last 12 months	0.00	1.00	678	9.6
Resides in the capital area	0.00	1.00	4152	58.6
***Continuous variables***	**Min**	**Max**	**Mean**	**SD**
Academic grades	4.00	32.00	21.59	5.41
Time spent with parents	2.00	10.00	6.46	2.05
Peer smoking	1.00	5.00	1.90	0.99
Peer drunkenness	1.00	5.00	2.00	1.07
Peer respect for smoking	1.00	5.00	1.91	1.05
Peer respect for alcohol use	1.00	5.00	2.30	1.10
Peer respect for cannabis use	1.00	5.00	1.69	1.00
Parental Education	−6.24	3.76	.01	2.70
Family financial status	1.00	7.00	3.52	1.05
School level peer smoking	1.00	4.00	1.90	0.30
School level peer drunkenness	1.00	3.33	2.00	0.27
School level peer cannabis use	0.00	55.56	25.20	11.55

### Dependent variables

Daily smoking, lifetime drunkenness, lifetime cannabis use comprised the dependent variables. Daily smoking was assessed with the question “How many cigarettes have you smoked during the last 30 days?” Response categories were 1 = None, 2 = Less than one cigarette per week, 3 = Less than one cigarette per day, 4 = 1–5 cigarettes per day, 5 = 6–10 cigarettes per day, 6 = 11–20 cigarettes per day, and 7 = More than 20 cigarettes per day. The scores were combined to form a dichotomized measure with 0 = None or less than daily, and 1 = daily. The questions forming the lifetime drunkenness and cannabis use were: “How often, if ever, in your lifetime have you a) got drunk and b) smoked hash/marijuana”. Response categories ranged from 1 = Never, 2 = Once, 3 = 2–5 times, 4 = 6–9 times, 5 = 10–19 times, 6 = 20–39 times, and 7 = 40 times or more often. The scores were collapsed to form dichotomized variables with 0 = Never, and 1 = Once or more often.

### Independent variables

#### Academic achievement

Academic achievement is an important marker for adolescent development and well-being [29]. It was assessed with four questions about the respondent’s performance in Icelandic, Mathematics, English and Danish (alternatively Norwegian or Swedish). These are the so called “unitary subjects” in Iceland and are compulsory for all students [30]. The Icelandic school system operates with a grading system on a 1–10 scale, where 5 or above constitutes a pass and below 5 a fail. Response categories ranged from 1 = Less than 4, 2 = About 4, 3 = About 5, 4 = About 6, 5 = About 7, 6 = About 8, 7 = About 9, and 8 = About 10. The scores were summed to form a scale with a range from 4 to 32 (Cronbach’s α = .80).

#### Time spent with parents

The amount of time adolescents spend with their parents, as opposed to what they actually do with them, has emerged as an important protective factor for substance use initiation and progression [18,19]. Time spent with parents was measured with two questions headed with “How well do the following apply to you: I spend time with my parents a) outside school hours on working days, and b) during weekends?” Responses range from 1 = Almost never, 2 = Seldom, 3 = Sometimes, 4 = Often, and 5 = Almost always. The two variables were summed to form a scale ranging from 2 to 10 (Cronbach’s α = .80).

#### Peer substance use

In line with the dependent variables peer substance use was measured with three questions headed with “How many of your friends do the following; a) smoke cigarettes, b) become drunk at least once per month, and c) smoke hash or marijuana”. Response categories range from 1 = None, 2 = A few, 3 = Several, 4 = Most, and 5 = Nearly all. Due to heavy positive skew the variable pertaining cannabis use was collapsed to form a dichotomized measure with 0 = None and 1 = Some. The variables relating to smoking and drunkenness were used untransformed. These three measures were also aggregated to the respective within-school mean in order to form a higher level measure. For the cannabis question this results in the school-level prevalence in knowing anyone that smokes hash or marijuana.

#### Peer respect for substance use

Peer respect for substance use was measured with three questions with the following heading: “How much do the following matter in order to gain respect in your peer group: a) to smoke cigarettes, b) to drink alcohol, and c) to smoke cannabis substances”. Response categories range from 1 = Decreases respect greatly, 2 = Decreases respect somewhat, 3 = Has no effect, 4 = Increases respect somewhat, and 5 = Increases respect greatly.

#### Gender

Gender was coded 1 for girls (51.5%) and 0 for boys.

#### Family structure

Family structure was measured with the question “Who lives in your home”: Response categories range from 1 = Both parents, 2 = Mother but not father, 3 = Father but not mother, 4 = Mother and her partner, 5 = Father and his partner, 6 = I live on my own, 7 = I live equally much but separately with my mother and father, 8 = I live with different arrangements. This variable was collapsed to form a dichotomized measure with 0 = Both parents, and 1 = Other forms.

#### Parental education

The educational background of parents remains one of the strongest predictors for adolescent well-being and development. Hence, in order to extract other influences it is an important control variable. Parental education was measured with two questions headed with “What is the highest level of education by your a) mother and b) father”. Responses range from 1 = Finished secondary school or less, 2 = Began high school or vocational school but did not graduate, 3 = Graduated from high school or vocational school, 4 = Began college but did not graduate, 5 = College graduate, 6 = Don’t know. These variables were mean-centered and combined to form a scale for parental education.

#### Family financial status

Family relative financial status is an important marker for substance use risk and adolescent development [31]. We measure family financial status with the question “If you think about the financial position of your family, how is it in comparison to other families in Iceland?” Response categories range from 1 = Much worse, 2 = Considerably worse, 3 = A little worse, 4 = About the same, 5 = A little better, 6 = Considerably better, and 7 = Much better.

#### School neighborhood

About 13% of the respondents in the study do not attend school in their home neighborhood. For analytical purposes we control for this discrepancy.

#### Residential moving and school change

Moving between neighborhoods/areas and changing schools can be stressful for adolescents and hence is a risk factor for substance use [31]. We control for such environmental change with the following questions: “Have you ever, during the last 12 months: a) changed schools, and b) moved between neighborhoods.” Response categories were 0 = No and 1 = Yes with 6.9% responding to have changed schools and 9.6% moving between neighborhoods during the last 12 months.

#### Place of residence

Approximately 60% of the population of Iceland resides within the greater capital area of Reykjavik, the country’s only urban center. Lifestyle patterns are in many ways different for adolescents residing inside and outside the capital area. Hence, it is a control variable in the study and is coded 1 = Resides in the capital area and 0 = Resides outside the capital area.

### Data analyses

Our data analyses were carried out with HLM 7.0 for multilevel logistic regression models with binary data [14,15]. All individual level variables are estimated with random effects and reported as such if statistically significant. First we ran the “empty” model without any predictor variables to assess the variance in the outcome variable that is attributable to the individual and school-levels and report the mean odds ratio (MOR) and intraclass correlation coefficients (ICC). We then proceeded to include the full models with individual level fixed effects and report any significant random effects, or unexplained variance between schools, in the mean odds of the independent variables, both in the intercept and predictor variables. We also report a calculation of explained variance using the formula outlined by Snijders and Bosker [14], (pp. 225-226) for multilevel logistic models, and significant cross-level interaction relationships for the substance use variables and school level peer use. The method of estimation is restricted penalized quasi-likelihood and all significance tests are based on robust standard errors.

## Results

Table 1 depicts the descriptive statistics for all study variables. Close to 7% of the study participants reported smoking on a daily bases and 28% had become drunk once or more often in their lifetime. In addition, almost 8% of the study sample admitted to using cannabis substances ever in their lives.

Table 2 shows the results from the multilevel analysis predicting daily smoking. The empty model reveals a little less than 10% of the variability in smoking being due to differences across school communities. The full model shows that individual level academic grades and time spent with parents are the strongest protective factors for smoking used in the study. For every one step increase academic grades the odds for daily smoking decrease by 6%. For every one step increase in time spent with parents the odds for daily smoking decrease by over 14%. As expected, the main risk factors for daily smoking are lifetime drunkenness (OR = 6.95) and lifetime cannabis use (OR = 3.54) but peer smoking and peer respect for smoking are also of importance. For every one step increase in peer smoking the odds for daily smoking increase over twofold and for every one step increase in peer respect for smoking the odds for daily smoking increases by about 85%. Additionally to these risk factors, school level peer smoking increases the odds for daily smoking by over 91% for each one step increase in the peer smoking measure. Only the slope for lifetime cannabis use differs across schools in the full model as indicated in the random effects.

**Table 2:** Multilevel logistic regression models with odds ratios and 95% confidence intervals predicting daily smoking

***Model***	**Empty**	**Full**			
*Individual level fixed effects*^†^		OR		95% CI for ORs	
Academic grades		0.940***		0.916-0.965	
Time spent with parents		0.856***		0.801-0.916	
Peer smoking		2.234***		1.921-2.600	
Peer respect for smoking		1.849***		1.630-2.097	
Lifetime drunkenness		6.949***		5.012-9.634	
Lifetime cannabis use		3.540***		2.583-4.850	
Gender		0.963		0.749-1.239	
Family structure		1.041		0.807-1.343	
Parental Education		0.968		0.929-1.008	
Attends school in home neighborhood		0.816		0.597-1.114	
Family financial status		0.962		0.867-1.069	
Resid. moving in the last 12 months		1.338		0.920-1.947	
Changed schools in the last 12 months		1.544*		1.026-2.324	
Resides in the capital area		0.872		0.660-1.153	
*School level fixed effects*^††^					
Peer smoking		1.914**		1.281-2.859	
*Random effects*		Var. comp.	SD	df	*χ*^2^
Lifetime cannabis use *u*_12_		0.394**	0.62	109	149.04
Intercept *u*_0_		0.130	0.36	108	120.99
*Model fit statistics*					
MOR	1.76				
ICC	0.096				
R^2^_dicho_		0.83			
−2 log likelihood	−9738.16	−8361.76			

† Based on Wald T and 6,791 df.

†† Based on Wald T and 139 df.

p < .05, ** p < .01, *** p < .001.

Table 3 shows the results from the multilevel analysis predicting lifetime drunkenness. The empty model reveals a little less than 5% of the variability in lifetime drunkenness being due to differences across school communities. As with smoking the full model shows that individual level academic grades and time spent with parents are the strongest protective factors for lifetime drunkenness included in the model. For every one step increase academic grades the odds for lifetime drunkenness decrease by 5%. For every one step increase in time spent with parents the odds for lifetime drunkenness decrease by close to 13%. As in the previous model the main risk factors for lifetime drunkenness are daily smoking (OR = 7.47) and lifetime cannabis use (OR = 8.03) but peer drunkenness and peer respect for alcohol use are also of importance. For every one step increase in peer drunkenness the odds for daily smoking increase by 2.4 and for every one step increase in peer respect for alcohol use the odds for lifetime drunkenness increases by over 3.5. Additionally to these risk factors, school level peer drunkenness increases the odds for lifetime drunkenness by about 52% for each one step increase in the peer drunkenness measure. In addition, the cross level interaction relationship between individual level peer respect for alcohol use and school level peer drunkenness indicates that the relationship between peer respect for alcohol use and risk of drunkenness is less in schools were peer drunkenness is common. Only the slope for family financial status differs across school communities in the full model as indicated in the random effects.

**Table 3:** Multilevel logistic regression models with odds ratios and 95% confidence intervals predicting lifetime drunkenness

***Model***	**Empty**	**Full**			
*Individual level fixed effects*^†^		OR		95% CI for ORs	
Academic grades		0.952***		0.940-0.964	
Time spent with parents		0.864***		0.837-0.892	
Peer drunkenness		2.423***		2.245-2.619	
Peer respect for alcohol use		3.538***		2.272-5.509	
Daily smoking		7.466***		4.465-11.227	
Lifetime cannabis use		8.032***		5.864-11.003	
Gender		1.396***		1.217-1.601	
Family structure		1.399***		1.225-1.598	
Parental Education		0.981		0.956-1.006	
Attends school in home neighborhood		0.942		0.771-1.152	
Family financial status		1.038		0.969-1.113	
Resid. moving in the last 12 months		0.984		0.736-1.316	
Changed schools in the last 12 months		0.973		0.688-1.378	
Resides in the capital area		0.808*		0.683-0.955	
*School level fixed effects*^††^					
Peer drunkenness		1.518**		1.129-2.042	
*Cross-level interaction effect*					
Peer respect for alcohol use*School level peer drunkenness		0.702***		0.565-0.872	
*Random effects*		Var. comp.	SD	df	*χ*^2^
Family financial status *u*_5_		0.012*	0.01	135	163.76
Intercept *u*_0_		0.064	0.25	134	188.72
*Model fit statistics*					
MOR	1.47				
ICC	0.047				
R^2^_dicho_		0.87			
−2 log likelihood	−9903.62	−10329.97			

† Based on Wald T and 6,513 df.

†† Based on Wald T and 139 df.

* p < .05, ** p < .01, *** p < .001.

Table 4 shows the results from the multilevel analysis predicting lifetime cannabis use. The empty model indicates that just over 5% of the variability in lifetime cannabis use is due to differences across school communities. The full model shows that being a girl and time spent with parents are the strongest protective factors for lifetime cannabis use in the study. For every one step increase in time spent with parents the odds for lifetime cannabis use decrease by about 11.5%. Also, being a girl is associated with considerably less risk in lifetime cannabis use (OR = 0.36). On the other hand the main risk factors for lifetime cannabis use are peer cannabis use (OR = 7.12), lifetime drunkenness (OR = 5.32), and daily smoking (OR = 3.82) but peer respect for cannabis use is also of importance. Different from the school community aggregate measures for peer smoking and peer drunkenness we do not find a significant association between school level peer cannabis use and odds of cannabis use. Only the random intercept differs significantly between school communities as shown in the random effects.

**Table 4:** Multilevel logistic regression models with odds ratios and 95% confidence intervals predicting lifetime cannabis use

***Model***	**Empty**	**Full**			
*Individual level fixed effects*^†^		OR		95% CI for ORs	
Academic grades		0.983		0.964-1.002	
Time spent with parents		0.885***		0.839-0.933	
Peer cannabis use		7.120***		5.365-9.449	
Peer respect for cannabis use		1.355***		1.228-1.495	
Daily smoking		3.824***		2.870-5.097	
Lifetime drunkenness		5.322***		3.996-7.089	
Gender		0.356***		0.283-0.448	
Family structure		1.084		0.874-1.344	
Parental Education		1.007		0.964-1.053	
Attends school in home neighborhood		1.025		0.761-1.382	
Family financial status		0.933		0.855-1.020	
Resid. moving in the last 12 months		1.132		0.761-1.682	
Changed schools in the last 12 months		1.344		0.948-1.906	
Resides in the capital area		0.920		0.704-1.201	
*School level fixed effects*^††^					
Peer cannabis use		1.009		0.995-1.024	
*Random effects*		Var. comp.	SD	df	*χ*^2^
Intercept *u*_0_		0.115**	0.34	138	180.79
*Model fit statistics*					
MOR	1.51				
ICC	0.053				
R^2^_dicho_		0.76			
−2 log likelihood	−9967.05	−10449.14			

† Based on Wald T and 6,791 df.

†† Based on Wald T and 139 df.

* p < .05, ** p < .01, *** p < .001.

## Discussion

Our findings show that peer smoking and alcohol use are not only related to such use between individuals during adolescence but also contextually through school communities. Not only do the odds for daily smoking increase by 2.2 for every point increase in individual-level peer smoking, they also increase an additional 91% for every point increase in school-level peer smoking. The same applies to lifetime drunkenness. The odds of lifetime drunkenness increase by 2.4 for every point increase individual-level peer drunkenness and by almost 52% for every point increase in school-level peer drunkenness. These findings also held for students that received high grades, spent a lot of time with their parents, belonged to a peer group where respect for substance use was low, and did not have any friends that smoked or became drunk. In contrast to other studies [22,23], we did not find the same contextual effects between individual and school-level peer cannabis use.

These findings provide an important addition to the currently limited evidence that adolescent substance use prevention efforts should target school and school communities directly in addition to individuals [18,19]. Furthermore, in our models we included a number of individual level variables (e.g., academic grades; time spent with parents; peer respect for substance use), that multilevel studies typically do not include. Another significant finding of our analyses were that the odds of daily smoking and lifetime drunkenness decreased continuously with rising academic achievement; but this relationship was not found for lifetime cannabis use. On a 28 point scale, the odds of daily smoking and lifetime drunkenness decreased by 5% and 6% respectively for each additional grade point. The same applies to time spent with parents. More time spent with parents decreased the odds of use for any of the three substances tested by 11-15% respectively. This mirrors our previous findings for time spent with parents [19]. These protective factors have also been found by others [32]. It is also noteworthy that the importance of peer respect for substance use is additional to individual and school level peer use on all three occasions. For every one-point increase in perceived peer respect for smoking, the odds for daily smoking increase by 85%. Similarly, each point increase in perceived peer respect for alcohol use increases the odds for lifetime drunkenness by over 3.5 times. Additionally, the importance of peer respect for alcohol use is less in schools were peer drunkenness is common, as indicated in the cross-level interaction variable. This is not a surprise as it is conceivable that respect for alcohol use should influence such use more if drunkenness is rare rather than if it is general and therefore not particularly daring among students.

The findings of the present study generally align with the prerequisites of the Icelandic model of substance use prevention [18-20]. This long-term approach has been utilized since 1997 and emphasizes community-level collaboration and dialogue between parental networks, school authorities, policy makers and practitioners. At the center of the approach is a focus on school communities as platforms for health education, collaboration and mutual exchange of knowledge and action, usually through the local schools. Over time this approach has led to a cultural shift in upbringing practices and general emphasis on contextual community effects with the peer group at the center of attention. Contextual relations, as shown in the present study, are associations of cultural aspect because they exert importance over and above individual level associations and, thus, impact on all individuals attending schools with higher prevalence of peer substance use, even though the individuals themselves may not be a part of substance using peer groups. The interpretational problem for such relations, on the other hand, resides, in part, in the current lack of theory that grasps both individual- and contextual effects. As Cook wrote ten years ago [16], p.154]: “Although descriptive [epidemiological] research is usually not highly prized, we consider it to be especially needed where existing theory is weak, as with theories about the joint influence of multiple contexts”.

Generally, in school surveys, between 3% and 7% of the variation in substance use is generated by the school-level with the bulk of the variance being within schools [33]. This does not mean that higher level data are not important; to the contrary, techniques of analysis such as multilevel methods have now allowed us to disentangle the potential importance of social contexts such as school-communities by simultaneously controlling for individual level relationships in our models. Research into other higher level contexts has found similar results as we do in the present study. For example, a study by Song et al. [34] suggested that community contexts are important for adolescent alcohol use, and Wright et al. [35] found the relative influences of neighborhoods and families accounted for up to 30% of the total variance in adolescent substance use.

This study has several limitations. First, the cross-sectional nature of the study precludes us from attributing cause and effect and therefore we cannot rule out other influences (e.g., selection and socialization) in addition to context. For example, we cannot rule out the possibility that adolescents in high prevalence schools initiated use before attending a particular school. As with all cross-sectional studies, the temporal order of events remains a largely unsolved problem. Nevertheless our findings add to a growing evidence base that contextual factors matter over and above individual level factors, even after taking account of multiple established individual-level components. Second, our analyses relied entirely on self-reports. We are therefore unable to guarantee responses without foundation to our survey questions. Third, the coding of the dependent variables in our study is somewhat unusual, particularly with regards to “daily smoking during last 30 days” and “lifetime drunkenness” rather than “any” smoking and alcohol use which is more common. This mismatch in time is particularly problematic in comparison to the peer substance use variables which do not include any reference to time. From an international comparative perspective, the dataset used in this study has rather low prevalence rates [36]. As already reported, school level variance tends to increase with higher prevalence rates [33]. Thus, it is possible that data from other countries would include a greater proportion of the total variance in substance use attributable to differences between schools and may provide additional understanding in these matters whilst coding the dependent variables in a different way.

Our study also has some strengths. First, we utilized a large and representative sample with high response rates. Second, our data come from a 15 year long line of cross-sectional studies that incorporate the same measures annually and have been rigorously tested for reliability and validity. Third, the nature of our statistical modeling enabled us to directly model the variance in school slopes instead of averaging it out as in traditional regression analyses therefore providing further evidence for the added value of higher level information in nested data structures.

## Conclusions

Findings of this study indicate that substance use prevention approaches among adolescents would benefit from focusing on school-communities as a whole and therefore all their students and not merely the individuals that seem likely to become substance users. A focus on ecologic, long-term primary prevention through local community health education and promotion, as successfully implemented in Iceland for over a decade and a half [18-20], may prove especially effective in reducing the frequency of adolescent substance use.

## Competing interests

The authors declare that they have no competing interests.

## Authors’ contributions

ALK conceived of the study, carried out statistical analyses and drafted multiple versions of the manuscript. IDS conceived of the study, participated in the study design and edited multiple versions of the manuscript. JPA reviewed multiple versions of the manuscript and participated in writing and editing the manuscript in its entirety. All authors read and approved the final manuscript.
